# Enhancing representation in cardiovascular trials: Lessons from Lp(a)FRONTIERS EXPANSION in United States Black and Hispanic patients with elevated lipoprotein(a) and established atherosclerotic cardiovascular disease

**DOI:** 10.1016/j.ajpc.2026.101467

**Published:** 2026-02-10

**Authors:** Keith C. Ferdinand, Fatima Rodriguez, Alok R. Amraotkar, Subha Venkataraman, Imran Ayaz, Oliver Antequera, Hui Cao, Wenyue Zhu, Jing Wang, Michael D. Shapiro

**Affiliations:** a Tulane University School of Medicine, John W Deming Department of Medicine, Section of Cardiology, New Orleans, LA, USA; b Stanford University School of Medicine, Cardiovascular Medicine and the Center for Digital Health, Stanford, CA, USA; c Novartis Pharmaceuticals Corporation, East Hanover, NJ, USA; d Novartis Pharmaceuticals UK Ltd, London, United Kingdom; e Wake Forest University School of Medicine, Center for Prevention of Cardiovascular Disease, Department of Cardiovascular Medicine, Winston-Salem, NC, USA

**Keywords:** Lipoprotein(a), Underrepresented diverse patients, ASCVD, pelacarsen, TQJ230

## Abstract

**Background:**

Black and Hispanic individuals are disproportionately impacted by elevated levels of lipoprotein(a) [Lp(a)] and atherosclerotic cardiovascular disease (ASCVD). Despite this, these populations are underrepresented in cardiovascular clinical trials. Herein, the strategies aimed to improve minority representation during the study design and recruitment phase of the Lp(a)FRONTIERS EXPANSION trial are described. Baseline characteristics and study attrition to date are also presented.

**Methods:**

Lp(a)FRONTIERS EXPANSION (NCT06267560) is a randomized, double-blind, phase 3b, placebo-controlled trial evaluating pelacarsen in United States (US) Black/African American and/or Hispanic individuals with elevated Lp(a) (≥125 nmol/L) and established ASCVD. Eligible individuals were randomized 2:1 to receive monthly subcutaneous injections of either pelacarsen 80 mg or placebo, for 12 months. Various design and operational strategies were employed to improve enrollment and retention under four key themes: study design, site selection and engagement, enrollment support, and barrier removal.

**Results:**

Overall, 422 patients were randomized to receive pelacarsen or placebo, with enrollment completed 1 year ahead of schedule. Patients were randomized from 103 sites across 21 US states/territories (including Puerto Rico); 8 sites recruited ≥10 patients. At data cut-off (7 October 2025), 64 patients had completed the trial. The mean±standard deviation age was 63.2 ± 9.1 years, 50.7% (214/422) of patients were male, and 68.0% (287/422) identified as Black/African American. Median (Q1, Q3) Lp(a) levels were 110.3 (79.4, 143.7) mg/dL (226.6 [165.8, 299.7] nmol/L).

**Conclusion:**

The Lp(a)FRONTIERS EXPANSION trial suggests that deliberate, culturally tailored design and operational strategies may improve cardiovascular trial participation by US minority populations. Results from Lp(a)FRONTIERS EXPANSION will provide critical insights into the efficacy and safety profile of pelacarsen in treating elevated Lp(a).

## Glossary

**Abbreviation Definition**
AE: Adverse event
apoB: Apolipoprotein B
ASCVD: Atherosclerotic cardiovascular disease
BMI: Body mass index
CV: Cardiovascular
eGFR: estimated Glomerular filtration rate
HbA1c: Glycated hemoglobin
HDL-C: High-density lipoprotein cholesterol
hsCRP: High-sensitivity c-reactive protein
ICF: Informed consent form
LDL-C: Low-density lipoprotein cholesterol
Lp(a): Lipoprotein(a)
PCR: Protein-creatinine ratio
Q: Quartile
QM: Once monthly
n: Number of patients with outcome
N: Number of patients in the group/number of sites
non–HDL-C: Non–high-density lipoprotein cholesterol
PCSK9: Proprotein convertase subtilisin/kexin type 9
SAE: Serious adverse event
SD: Standard deviation
SoC: Standard-of-care
ULN: Upper limit of normal
US: United States
VLDL-C: Very‒low-density lipoprotein cholesterol

## 1 Introduction

Elevated lipoprotein(a) [Lp(a)] is a common, genetic, causal, and independent cardiovascular disease risk factor [[1], [2], [3]]. It is estimated that approximately one in five individuals in the general population and one in four individuals with atherosclerotic cardiovascular disease (ASCVD) have elevated Lp(a), which can be observed worldwide across all racial and ethnic groups [4,5]. Lp(a) testing rates in the United States (US) remain extremely low, even among insured individuals, [6] with <1 % of patients receiving Lp(a) testing [[6], [7], [8], [9]]. There is distinct heterogeneity in Lp(a) levels [2,[10], [11], [12]]; people of sub-Saharan Africa/Black background are reported to have the highest median levels of Lp(a) and the highest prevalence of elevated Lp(a) [2,4,5,13]. US Hispanic individuals have also reported elevated median levels of Lp(a), [10,11] with significant heterogeneity based on country of origin [11]. Additionally, Black and Hispanic adults are disproportionately impacted by cardiovascular disease, [[14], [15], [16], [17], [18]] as well as cardiovascular-related mortality [[18], [19], [20]]. Despite these observations, these populations have low rates of Lp(a) testing and remain underrepresented in cardiovascular clinical trials [8,12,[21], [22], [23]].

Inclusion of historically underrepresented, ethnically diverse populations in clinical trials is paramount in promoting insights into disease demographics and the evaluation of the effectiveness and safety of potential treatments [11,24,25]. However, social determinants and systemic barriers to healthcare access can impact clinical trial recruitment in these populations [16]. These include adverse historical policies, uncontrolled comorbidities that are generally excluded from clinical trials, lack of awareness or access to clinical trials, cultural differences, and other socioeconomic factors [16]. Thus, a multi-faceted approach to trial recruitment is necessary, and the criticality of the role of study design, recruitment strategy, site selection, and resource allocation must be recognized [26,27]. Representation imbalance in cardiovascular trials is well-documented and the success of planned preparedness to address systemic barriers has shown to be effective many times [[26], [27], [28]].

There is growing research describing techniques and strategies that may help improve participation of various populations, and the documentation of benefits or harms, in cardiovascular clinical trials [23,[29], [30], [31]]. The overall objective of the current analysis is to comprehensively describe the strategies employed to mitigate systemic barriers to clinical trial participation during the study design and recruitment phase of the Lp(a)FRONTIERS EXPANSION trial. Additionally, baseline characteristics and study attrition to date are presented.

## 2 Methods

### 2.1 Study design and population

Lp(a)FRONTIERS EXPANSION (NCT06267560) is a randomized, double-blind, phase 3b, placebo-controlled trial in US Black/African American and/or Hispanic individuals (including Puerto Rico) with elevated Lp(a) (≥125 nmol/L) and established ASCVD. Lp(a)FRONTIERS EXPANSION aims to assess the efficacy and safety of pelacarsen in lowering Lp(a) after 12 months of treatment. The inclusion criterion of Lp(a) ≥125 nmol/L as a risk-enhancing factor is consistent with the American College of Cardiology/American Heart Association recommendations for high cardiovascular risk assessment [32]. Additionally, the trial aimed to enroll ≥60 % Black patients overall, as per protocol requirements. The Lp(a)FRONTIERS EXPANSION trial is distinct from the primary phase 3 clinical trial, Lp(a)HORIZON (NCT04023552), a large cardiovascular outcomes trial aiming to evaluate the efficacy and safety of pelacarsen in patients with elevated Lp(a) (≥70 mg/dL) and established cardiovascular disease [33].

**Fig. 1:**
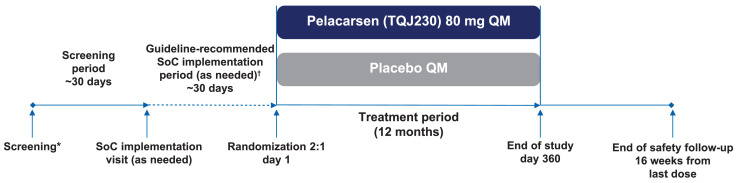
Study design of the Lp(a)FRONTIERS EXPANSION clinical trial. *The screening visit was optionally conducted either as a one-day visit (i.e., Part 1 and Part 2 on the same visit day) or on two days (Part 1 and Part 2 on different days). Operational strategies were designed and integrated into the study design of the trial (i.e. prior to the beginning of screening). All sites were encouraged to implement these strategies during the recruitment phase when sites were being activated. **^†^**The guideline-recommended SoC implementation period is only needed for patients who require a SoC treatment initiation for other cardiovascular risk factors (elevated LDL-C levels, elevated blood pressure, diabetes). LDL-C, low-density lipoprotein cholesterol; SoC, standard of care; QM, once monthly.

The Lp(a)FRONTIERS EXPANSION trial includes three periods: screening, treatment, and safety follow-up (Fig. 1). The screening period was approximately 30 days, with the option of splitting the screening visit into 2 separate days. This option was provided to limit the amount of time spent by patients on site, and to ensure they fulfilled the key Lp(a) entry criteria before going through other extensive protocol-required assessments. This screening visit during the screening period was followed by a guideline-recommended standard of care (SoC) implementation visit for patients who required initiation or optimization of treatment for other modifiable cardiovascular risk factors (i.e. elevated low-density lipoprotein cholesterol [LDL-C], diabetes, and high blood pressure). Considering the disparities in healthcare access by these underrepresented populations, patients were offered free SoC treatment, which was initiated according to local practice/guidelines within 30 days from the screening visit and at least 30 days prior to randomization. Following screening and SoC treatment implementation if required, eligible patients were randomized in a 2:1 ratio to receive monthly subcutaneous injections of pelacarsen 80 mg or placebo for 12 months, increasing the chance of receiving investigational treatment. Patients (or their caregivers) have the option of self-administering the study drug at home, if desired, after adequate training on site; otherwise, the study drug is administered by site staff. All randomized patients will have a safety follow-up conducted at least 16 weeks after the last administration of study treatment.

This trial complies with the principles outlined in the most recent revision of the Declaration of Helsinki, the International Conference on Harmonization guidelines for Good Clinical Practice, and all relevant local laws and regulations. The protocol received approval from local institutional review boards, and all patients gave written informed consent before any trial-specific procedures took place.

### 2.2 Trial endpoints

The primary endpoint of the trial is the change in log-transformed Lp(a) from baseline to week 52. Secondary endpoints include the safety and tolerability of pelacarsen, assessed by the incidence of treatment-emergent adverse events (AEs)/serious adverse events (SAEs), AEs/SAEs leading to treatment or study discontinuations, or changes in safety laboratory measures, vital signs, and electrocardiogram measurements.

### 2.3 Operational strategies employed

**Fig. 2:**
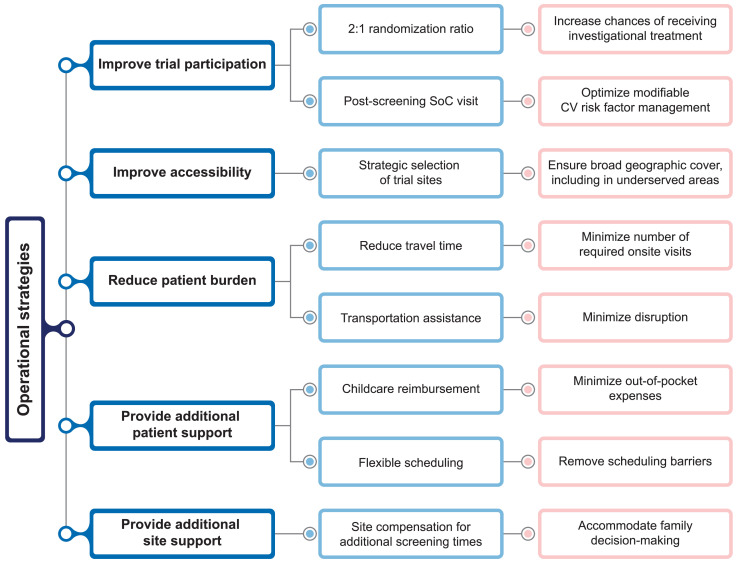
An overview of key operational strategies used in the Lp(a)FRONTIERS EXPANSION trial. CV, cardiovascular; SoC, standard of care.

Several key operational strategies were purposefully integrated into the trial besides the study design (as outlined above) to enhance trial enrollment and improve patient retention based on common barriers (Fig. 2). Sustained efforts were consistently incorporated from the trial design stage to all trial implementation phases. An in-person investigator meeting was organized during the trial recruitment phase to facilitate investigator training and trial updates. The integrated agenda and interactive topics provided an opportunity for knowledge sharing across sites.

### 2.4 Site selection and engagement

Site selection strategies were developed to combat common trial recruitment barriers which are summarized in Table 1. These strategies aimed to include a broad geographic coverage (including underserved areas), increase site engagement, and improve retention.

**Table 1:** Site selection and engagement operational strategies to combat common trial recruitment barriers employed in the Lp(a)FRONTIERS EXPANSION trial.

Strategy type	Description
Site strategies	• Sites were strategically chosen to include those in underserved areas with the aim to improve accessibility, reduce travel time for patients and to reflect the diversity with the US Hispanic population
	• Sites were compensated for any additional screening time to accommodate patients who involved families in decision-making
Patient strategies	• Post-screening SoC visit for patients who required cardiovascular risk factors other than Lp(a) to be optimized with treatment prior to randomization
	• Transportation assistance
	• Childcare reimbursement
	• Flexible scheduling
	• Involving families in the decision-making process
	• Minimized number of required on-site visits
Consent process and visit strategies	• At-length discussions on trial requirements at the time of consent, and improved patient awareness of time requirements and trial timelines
	• Coordinating office follow-up visits with trial visits to ensure visits are not missed, making it more convenient for patients
	• Creating a tracker for monitoring patient visit schedule and contacting patients by phone the day before their appointment as a reminder
	• Providing 24-hour access to a dedicated research phone number
Site selection and support strategies	• A designated vendor was assigned to help set up sites which had not previously participated in clinical trials run by the sponsor
	• Rural and community-based sites with a high number of minority populations were selected
	• A broad geographic coverage across the US (including Puerto Rico) was employed, with a representative range of principal investigators

Lp(a), lipoprotein(a); SoC, standard of care; US, United States.

### 2.5 Enrollment support

Trial sites and investigators were encouraged to pre-identify individuals that may be potentially suitable for trial enrollment, which was anticipated to expedite the recruitment process at these sites. Culturally sensitive approaches (e.g., language adaptations, including medical terminology, consideration of family decision-making processes, inclusion of investigators from the same communities etc.) were employed to explain trial aspects between potential patients and trial staff. One such method was to approach patients as continued partners in their own health outcomes, rather than ‘subjects’ in a clinical trial. Education on the benefits of Lp(a) testing was also provided in information brochures and patient-facing flyers.

### 2.6 Barrier removal

With broader geographic coverage, the overall travel time to sites is reduced for patients. Patients were provided with a stipend and a smooth reimbursement process to compensate them for their time and expenses associated with trial participation. If required, patients and their caregivers were provided with transportation to the trial sites, as well as childcare support services. Tailored educational materials for both patients and caregivers were developed to improve patient understanding of the trial and participation procedures.

### 2.7 Statistical analysis

Based on power calculations, approximately 400 patients were planned to be enrolled and randomly assigned in a 2:1 ratio to receive pelacarsen, or placebo. The planned sample size will provide ≥99 % power to detect a ‒1.28 log-transformed placebo-adjusted mean change from baseline for pelacarsen, based on a one-sided, two-sample *t*-test with a significance level of 0.025, assuming a 0.634 standard deviation (SD) of the placebo-adjusted mean change from baseline. This sample size will also guarantee a high probability of observing at least 1 occurrence of an adverse event with a true incidence rate of 1 %.

Randomization to study treatment was performed via interaction response technology with stratification based on race (Black/African American: yes vs. no) and ethnicity (US Hispanic: yes vs. no) at screening. All patients and clinical trial staff were blinded to study treatment.

The primary efficacy endpoint of change from baseline in log-transformed Lp(a) concentration at week 52 will be analyzed using a restricted maximum likelihood-based mixed model for repeated measures with fixed effects for treatment, visit, baseline log-transformed Lp(a), stratification factor, and the treatment-by-visit interaction. Visit will be included in the model as a categorical variable along with the treatment-by-visit interaction. This analysis will be conducted on the full analysis set, which comprises all patients to whom study treatment has been assigned by randomization, except for those who have not been qualified for this (and have therefore not received any study drug) but have been inadvertently randomized into the trial.

The summary of safety data will be presented by treatment group in the safety set, which includes all patients who received at least one dose of study treatment. No formal inferential testing will be performed, and summaries will be descriptive.

## 3 Results

### 3.1 Recruitment

**Fig. 3:**
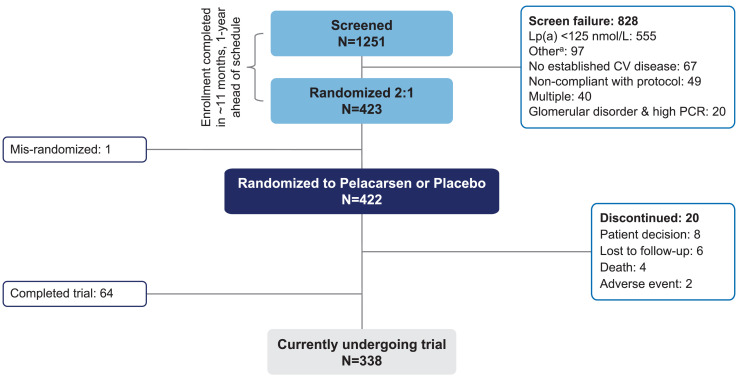
Patient disposition in the Lp(a)FRONTIERS EXPANSION trial at data cut-off (7 October 2025). ^a^Other included the following reasons: Unwanted symptoms present (*n* = 20), condition interfering inclusion (*n* = 13), low platelet count prior to day 1 (*n* = 12), confirmed virology test (*n* = 11), unwanted procedure performed (*n* = 6), uncontrolled disorders present (*n* = 6), low eGFR value prior to day 1 (*n* = 5), age not between 18 and 80 years (*n* = 4), total bilirubin ≥1.5x ULN (*n* = 4), no ICF/ICF date after screening (*n* = 4), uncontrolled hypertension (*n* = 3), no stable PCSK9 inhibitor dose (*n* = 3), other investigational drug use (*n* = 2), history of malignancy (*n* = 2), no optimal LDL-C lowering regimen (*n* = 1), and confirmed pregnancy or nursing (*n* = 1). CV, cardiovascular; eGFR, estimated glomerular filtration rate; ICF, informed consent form; LDL-C, low-density lipoprotein cholesterol; Lp(a), lipoprotein(a); N, number of patients in the group; n, number of patients with outcome; PCR, protein-creatinine ratio; PCSK9, proprotein convertase subtilisin/kexin type 9; ULN, upper limit of normal.

**Fig. 4:**
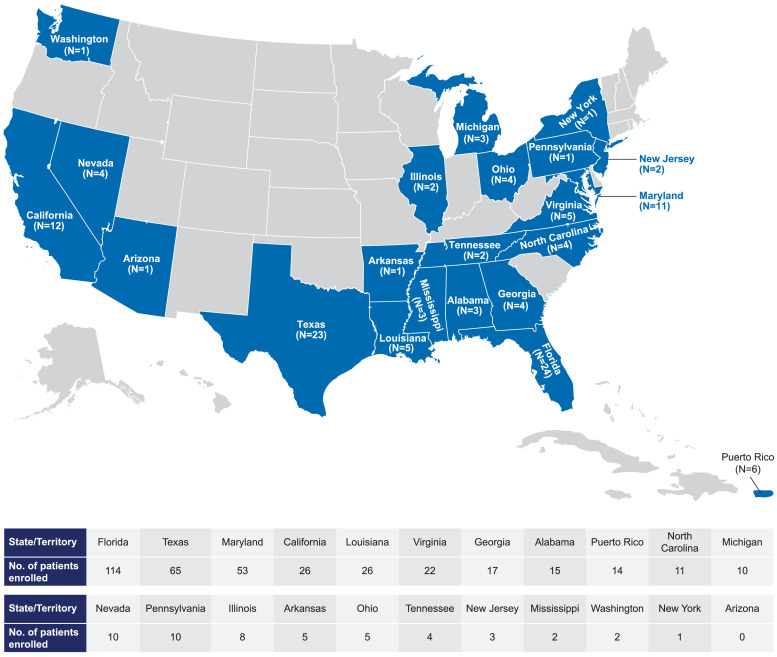
A map detailing the locations of trial sites which screened patients and the number of patients enrolled. Blue shading indicates where there was at least one site which screened patients for the Lp(a)FRONTIERS EXPANSION trial. Each state (or territory in the case of Puerto Rico) is marked with its name as well as the number of sites listed. The legend below the map indicates the number of patients randomized from each state/territory from all sites within that state/territory.

Over the course of approximately 11 months, 1251 individuals were screened for eligibility, with 828 being ineligible to participate, resulting in a screen failure rate of 66.2 % (828/1251). The most common reason for this was Lp(a) <125 nmol/L (67.0 % [555/828]). A total of 423 patients who met the eligibility criteria were randomized, with one mis-randomization, leaving 422 patients randomized to either the pelacarsen arm or the placebo arm (Fig. 3). The recruitment goal (recruiting approximately 400 patients over approximately 2 years) was reached a full year ahead of the planned time for recruitment. A total of 122 sites across 22 states/territories (including Puerto Rico) underwent screening for the trial and patients were randomized from 103 sites across 21 states/territories in the US (including Puerto Rico) (Fig. 4); 8 sites recruited ≥10 patients, 21 sites recruited 6–9 patients, and 35 sites recruited 3–5 patients.

N, number of sites.

At data cut-off (7 October 2025), a total of 338 patients were participating in the ongoing trial, 64 patients had completed the trial, and 20 patients had discontinued the trial, primarily due to patient decision (*n* = 8) (Fig. 3).

### 3.2 Baseline characteristics

Of the 422 patients who were randomized and received treatment, the mean±SD age was 63.2 ± 9.1 years, with 50.7 % (214/422) of patients being male, a near-equal sex ratio (Table 2). Overall, 68.0 % (287/422) of patients identified as Black/African American and 36.0 % (152/422) as Hispanic or Latino (Table 2). The overall median (Q1, Q3) Lp(a) was 110.3 (79.4, 143.7) mg/dL (226.6 [165.8, 299.7] nmol/L). Myocardial infarction (46.2 % [195/422]) was the most frequent prior event, with approximately 1 in 5 patients having a prior ischemic stroke (20.4 % [86/422]), and approximately 1 in 4 patients having peripheral artery disease (23.9 % [101/422]). A total of 60 (14.2 %) patients entered the SoC implementation period, with 35 (8.3 %) having their treatment initiated or optimized. Female patients had higher median (Q1, Q3) baseline Lp(a) levels versus male patients, as observed in both mg/dL and nmol/L measurement units (120.9 [86.2, 159.7] versus 99.4 [74.0, 133.4] mg/dL and 248.3 [178.1, 321.6] versus 208.5 [158.0, 276.6] nmol/L). Similarly, Black patients had higher median (Q1, Q3) baseline Lp(a) levels versus non-Black patients (114.5 [79.4, 147.0] versus 100.9 [78.8, 135.2] mg/dL and 235.5 [167.6, 308.1] versus 205.4 [164.3, 275.1] nmol/L).

**Table 2:** Baseline characteristics of patients enrolled in the Lp(a)FRONTIERS EXPANSION trial overall, and stratified by sex and race.

Characteristic	Overall (*N* = 422)	Sex		Race	
		Male (*N* = 214)	Female (*N* = 208)	Black (*N* = 287)	Non-Black (*N* = 135)
Age, mean±SD, years	63.2 ± 9.1	63.0 ± 8.9	63.4 ± 9.4	63.2 ± 9.3	63.1 ± 8.8
Sex, n ( %)					
Male	214 (50.7)	214 (100.0)	0 (0.0)	127 (44.3)	87 (64.4)
Female	208 (49.3)	0 (0.0)	208 (100.0)	160 (55.7)	48 (35.6)
Race, n ( %)					
White	125 (29.6)	81 (37.9)	44 (21.2)	0 (0.0)	125 (92.6)
Black/African American	287 (68.0)	127 (59.3)	160 (76.9)	287 (100.0)	0 (0.0)
Other	10 (2.4)	6 (2.8)	4 (1.9)	0 (0.0)	10 (7.4)
Ethnicity, n ( %)					
Hispanic or Latino	152 (36.0)^a^	95 (44.4)^b^	57 (27.4)^c^	21 (7.3)^d^	131 (97.0)^e^
Not Hispanic or Latino	270 (64.0)	119 (55.6)	151 (72.6)	266 (92.7)	4 (3.0)
Race and ethnicity (stratification), n ( %)					
Black and non-Hispanic	272 (64.5)	120 (56.1)	152 (73.1)	267 (93.0)	5 (3.7)
Non-Black and Hispanic	127 (30.1)	81 (37.9)	46 (22.1)	3 (1.0)	124 (91.9)
Black and Hispanic	23 (5.5)	13 (6.1)	10 (4.8)	17 (5.9)	6 (4.4)
Smoking history, n ( %)					
Never	203 (48.1)	93 (43.5)	110 (52.9)	129 (44.9)	74 (54.8)
Current	90 (21.3)	46 (21.5)	44 (21.2)	71 (24.7)	19 (14.1)
Former	128 (30.3)	75 (35.0)	53 (25.5)	87 (30.3)	41 (30.4)
Missing	1 (0.2)	0 (0.0)	1 (0.5)	0 (0.0)	1 (0.7)
BMI, mean±SD, kg/m^2^	31.3 ± 6.4	30.2 ± 5.9	32.4 ± 6.8	31.4 (6.8)	31.1 ± 5.7
Systolic blood pressure, mean±SD, mmHg	126.4 ± 14.4	126.2 ± 13.7	126.6 ± 15.0	126.9 ± 14.9	125.3 ± 13.1
Diastolic blood pressure, mean±SD, mmHg	75.2 ± 9.7	75.8 ± 9.6	74.6 ± 9.8	75.3 ± 10.1	75.1 ± 8.9
Lp(a), median (Q1, Q3), nmol/L	226.6 (165.8, 299.7)	208.5 (158.0, 276.6)	248.3 (178.1, 321.6)	235.5 (167.6, 308.1)	205.4 (164.3, 275.1)
Lp(a), median (Q1, Q3), mg/dL	110.3 (79.4, 143.7)	99.4 (74.0, 133.4)	120.9 (86.2, 159.7)	114.5 (79.4, 147.0)	100.9 (78.8, 135.2)
Lipids, mean±SD, mmol/L					
Total cholesterol	4.0 ± 1.1	3.8 ± 1.1	4.2 ± 1.1	4.0 ± 1.1	4.1 ± 1.2
Triglycerides	1.3 ± 0.7	1.3 ± 0.7	1.3 ± 0.7	1.1 ± 0.6	1.6 ± 0.8
LDL-C	2.3 ± 0.9	2.2 ± 0.9	2.4 ± 1.0	2.2 ± 0.9	2.3 ± 1.0
Non‒HDL-C	2.7 ± 1.1	2.6 ± 1.1	2.8 ± 1.1	2.6 ± 1.0	2.9 ± 1.1
HDL-C	1.4 ± 0.4	1.2 ± 0.4	1.5 ± 0.4	1.4 ± 0.4	1.2 ± 0.4
VLDL-C	0.6 ± 0.3	0.6 ± 0.3	0.6 ± 0.3	0.5 ± 0.3	0.7 ± 0.4
ApoB, mean±SD, g/L	0.7 ± 0.3	0.7 ± 0.3	0.8 ± 0.3	0.7 ± 0.2	0.8 ± 0.3
Baseline PCSK9 inhibitor usage, n ( %)	30 (7.1)	11 (5.1)	19 (9.1)	23 (8.0)	7 (5.2)
HbA1c, mean±SD, %	6.5 ± 1.3	6.6 ± 1.5	6.4 ± 1.2	6.4 ± 1.2	6.8 ± 1.4
Plasma glucose, mean±SD, mmol/L	6.8 ± 2.9	7.0 ± 3.0	6.6 ± 2.8	6.4 ± 2.6	7.5 ± 3.4
hsCRP, mean±SD, mg/L	5.0 ± 10.6	4.8 ± 11.8	5.2 ± 9.2	5.2 ± 10.5	4.7 ± 10.9
Event/disease history, n ( %)					
Myocardial infarction	195 (46.2)	115 (53.7)	80 (38.5)	124 (43.2)	71 (52.6)
Ischemic stroke	86 (20.4)	36 (16.8)	50 (24.0)	71 (24.7)	15 (11.1)
Peripheral artery disease	101 (23.9)	47 (22.0)	54 (26.0)	75 (26.1)	26 (19.3)
Baseline statin intensity, n ( %)					
High	318 (75.4)	167 (78.0)	151 (72.6)	216 (75.3)	102 (75.6)
Medium	46 (10.9)	23 (10.7)	23 (11.1)	33 (11.5)	13 (9.6)
Low	19 (4.5)	7 (3.3)	12 (5.8)	13 (4.5)	6 (4.4)
None	39 (9.2)	17 (7.9)	22 (10.6)	25 (8.7)	14 (10.4)

Data presented are based on snapshot of the database performed on October 7, 2025.

^a^Hispanic origin/background included: Dominican (*n* = 4), Central American (*n* = 6), Cuban (*n* = 67), Mexican (*n* = 26), Puerto Rican (*n* = 26), South American (*n* = 15), and Other (*n* = 8). ^b^Hispanic origin/background included: Dominican (*n* = 2), Central American (*n* = 4), Cuban (*n* = 41), Mexican (*n* = 20), Puerto Rican (*n* = 15), South American (*n* = 8), and Other (*n* = 5). ^c^Hispanic origin/background included: Dominican (*n* = 2), Central American (*n* = 2), Cuban (*n* = 26), Mexican (*n* = 6), Puerto Rican (*n* = 11), South American (*n* = 7), and Other (*n* = 3). ^d^Hispanic origin/background included: Dominican (*n* = 1), Central American (*n* = 1), Cuban (*n* = 12), Puerto Rican (*n* = 5), and Other (*n* = 2). ^e^Hispanic origin/background included: Dominican (*n* = 3), Central American (*n* = 5), Cuban (*n* = 55), Mexican (*n* = 26), Puerto Rican (*n* = 21), South American (*n* = 15), and Other (*n* = 6).

apoB, apolipoprotein B; BMI, body mass index; HbA1c, glycated hemoglobin; HDL-C, high-density lipoprotein cholesterol; hsCRP, high-sensitivity c-reactive protein; LDL-C, low-density lipoprotein cholesterol; Lp(a), lipoprotein(a); n, number of patients with outcome; N, number of patients in the group; non–HDL-C, non–high-density lipoprotein cholesterol; PCSK9, proprotein convertase subtilisin/kexin type 9; Q, quartile; SD, standard deviation; VLDL-C, very low-density lipoprotein cholesterol.

## 4 Discussion

The Lp(a)FRONTIERS EXPANSION trial is a phase 3b clinical trial designed to evaluate the efficacy and safety of pelacarsen in US Black/African American and Hispanic populations with elevated Lp(a) (≥125 nmol/L) and established ASCVD. This trial will also further expand the understanding of pelacarsen’s efficacy and safety in populations which are underrepresented in the Lp(a)HORIZON pivotal pelacarsen trial [33]. Lp(a)FRONTIERS EXPANSION was designed to mitigate the systemic barriers to clinical trial participation in minority populations in the US and may provide a valuable framework to help improve diversity in cardiovascular clinical trials. A lack of demographic representation impacts the understanding of molecular mechanisms of novel therapeutics and risks the paucity of their generalized applicability [27,34]. Addressing barriers to research participation may improve enrollment of these populations in cardiovascular clinical trials, ensuring that promising therapies are evaluated in patient cohorts that reflect those most affected by elevated Lp(a). For many years, efforts have been made to improve both the diversity and minority representation in cardiovascular clinical trials and research studies, indicating that it is imperative to improve diversity and generalizability of results [15,23,35]. However, despite these efforts, enrollment of minority populations has not improved over time [22,36].

In Lp(a)FRONTIERS EXPANSION, 68 % of enrolled patients were Black and 36 % were Hispanic or Latino, representing a significant improvement from both historical patterns of cardiovascular clinical trials and more recent Lp(a) clinical trials, where just ∼2‒4 % of enrollees were reported as Black and ∼2‒15 % were reported as Hispanic or Latino [33,[37], [38], [39]]. The representation of Black patients in National Institute of Health‒funded cardiovascular trials between 2000 and 2019 remained unchanged and very low, with 46 % recruiting <25 % Black patients. Additionally, only 21 % of trials during this period defined a recruitment target for underrepresented groups [22]. Similarly, an analysis which examined a 10-year trend of large cardiometabolic clinical trials reported that 81 % of trial participants were White adults, with no trend for improved enrollment of minority populations over time [36]. Lp(a)FRONTIERS EXPANSION diverges from these patterns and highlights that deliberately tailored implementation strategies may improve enrollment of these minority populations. Study design and trial conceptualization are the earliest and most profound building blocks of proper population representation in clinical studies [26,27,34]. Sustained efforts through all phases of the clinical trial, including preparedness for potential barriers, precede increased study participation in chronic diseases [26,27,34]. Given the enrollment success of Lp(a)FRONTIERS EXPANSION (completed approximately 1-year ahead of schedule), the long-term planning and focused efforts are noted in this current study. Although not directly measured in this study, the relationship between these systemwide efforts and high-quality patient enrollment is highly probable.

In recent years, there has been a greater push towards developing strategies that improve the inclusion of broader populations in cardiovascular research, with many groups publishing potentially successful study design considerations to help improve enrollment [16,[29], [30], [31]]. As aforementioned, despite being disproportionately affected by elevated Lp(a) levels and the resulting increased risk of ASCVD and cardiovascular-related mortality, Black/African American and Hispanic individuals remain significantly underrepresented in clinical trials; logistical obstacles and barriers, historical mistreatment of minority populations, and a lack of diverse trial investigators have been previously proposed as potential contributing factors to this misrepresentation [40,41]. In alignment with prior reports, [5,42] in Lp(a)FRONTIERS EXPANSION, median Lp(a) levels were higher in Black (114.5 mg/dL and 235.5 nmol/L) versus non-Black (100.9 mg/dL and 205.4 nmol/L) patients and in females (120.9 mg/dL and 248.3 nmol/L) versus males (99.4 mg/dL and 208.5 nmol/L), reinforcing the need to include these populations in trials investigating Lp(a)-targeted therapies. The elevated median Lp(a) levels and minimal variation in baseline characteristics across study groups suggest that recruitment efforts in an underrepresented population enhanced both the clinical and scientific relevance of the study.

The design and operational strategies implemented in Lp(a)FRONTIERS EXPANSION aimed at overcoming known barriers to clinical trial participation in US minority populations. These included a broad site selection in underserved areas, support from investigators from the same communities, trial participation-related barrier removal (e.g. transportation, stipends, childcare), study design considerations (e.g. SoC visit, optional self-administration of the study drug, visit flexibility), and enrollment support (e.g. pre-identification of patients). Although establishing a direct link between these strategies and the reduction of barriers to research participation is challenging, inference is highly probable given that the trial achieved its full enrollment goals (>400 patients) in approximately 11 months, a year earlier than originally planned. Moreover, the cohort exhibited a near-equal sex ratio, overcoming the frequently reported underrepresentation of women in cardiovascular trials [29,36]. While these outcomes are promising, implementing these strategies involves some implicit financial considerations in trial design. While some low-resource strategies such as the 2:1 randomization ratio, strategic selection of trial sites, consenting process, and flexible scheduling may be easily implemented, high-resource strategies including the free SoC visit, transportation assistance, childcare reimbursement, and stipends may be more challenging to implement outside of industry-sponsored trials. Nevertheless, these findings collectively suggest that well-planned, culturally tailored study design and operational strategies not only help recruit more diverse patient groups but also help to accelerate recruitment. While more progress is needed, these results offer hopeful strategies to address systemic barriers to research participation among underrepresented populations in cardiovascular trials and set the stage for future efforts to enhance participation and outcomes.

### 4.1 Limitations

Several operational strategies were employed in the Lp(a)FRONTIERS EXPANSION trial with the aim of improving enrollment of minority populations. However, due to the design of the trial, the effectiveness of specific strategies on enrollment cannot be determined, reflected by the exploratory nature of this analysis. Additionally, the lack of a comparator group for the operational strategies means that causality cannot be determined. Furthermore, although operational strategies were employed across all sites, the lack of site level comparisons is a limitation. Lastly, high resource operational strategies (e.g. childcare support, transport assistance) likely limit the scalability and generalizability of these operational strategies beyond industry-sponsored trials.

## 5 Conclusions

Underrepresentation of minorities in clinical trials despite being at higher risk for cardiovascular disease limits the generalizability of findings and potentially exacerbates health disparities. The Lp(a)FRONTIERS EXPANSION randomized, double-blind, phase 3b, multicenter trial suggests that purposefully tailored operational strategies may positively improve participation in cardiovascular clinical trials in minority populations in the US. The findings from the Lp(a)FRONTIERS EXPANSION trial will provide important data to strengthen the evidence base regarding the efficacy and safety of pelacarsen in Black/African American and Hispanic individuals with elevated Lp(a) and established ASCVD.

## Funding sources

This trial was sponsored by Novartis Pharma AG, Basel, Switzerland.

## Data availability

Novartis is committed to sharing access to patient-level data and supporting clinical documents from eligible studies with qualified external researchers. These requests are reviewed and approved by an independent review panel on the basis of scientific merit. All data provided are anonymized to respect the privacy of patients who have participated in the trials in line with applicable laws and regulations. The availability of these trial data is in accordance with the criteria and the process described at https://www.clinicalstudydatarequest.com.

## Ethics approval/statement

The study protocol and all amendments for this trial were reviewed by the Independent Ethics Committee or Institutional Review Board for each participating center. The study was done according to The International Conference on Harmonization Guidelines for Good Clinical Practice that have their origin in the Declaration of Helsinki. Written informed consent was obtained from each patient during the screening visit and before any study-specific procedure was done.

## CRediT authorship contribution statement

**Keith C. Ferdinand:** Writing – review & editing, Methodology, Investigation, Conceptualization. **Fatima Rodriguez:** Writing – review & editing, Methodology, Investigation, Conceptualization. **Alok R. Amraotkar:** Writing – review & editing, Project administration. **Subha Venkataraman:** Writing – review & editing, Writing – original draft, Project administration, Methodology, Conceptualization. **Imran Ayaz:** Writing – review & editing, Project administration, Formal analysis, Data curation. **Oliver Antequera:** Writing – review & editing, Project administration, Formal analysis, Data curation. **Hui Cao:** Writing – review & editing, Writing – original draft, Project administration, Methodology, Conceptualization. **Wenyue Zhu:** Writing – review & editing, Formal analysis, Data curation. **Jing Wang:** Writing – review & editing, Methodology, Formal analysis, Data curation, Conceptualization. **Michael D. Shapiro:** Writing – review & editing, Methodology, Investigation, Conceptualization.

## Declaration of competing interest

**Keith C. Ferdinand** is a member of the OCEANIC-AF Steering Committee and is a consultant for Novartis, Medtronic, Eli Lilly, Boehringer Ingelheim, and Janssen. **Fatima Rodriguez** reports equity from Carta Healthcare and HealthPals; and has received consulting fees from Novartis, Novo Nordisk, Esperion Therapeutics, Movano Health, Kento Health, Edwards, Arrowhead Pharmaceuticals, Cleerly, Irhythm, and HeartFlow. **Alok Amraotkar, Subha Venkataraman, Imran Ayaz, Oliver Antequera, Hui Cao, Wenyue Zhu**, and **Jing Wang** are employees and/or stockholders of Novartis. **Michael D. Shapiro** has the following research grants (to his institution) - Amgen, Boehringer Ingelheim, Cleerly, Novartis, Ionis, Lilly, Merck, New Amsterdam; serves on the following Scientific Advisory Boards: Amgen, Ionis, Esperion, Novartis, Regeneron, Lilly, Merck; and serves as a Consultant to: Tourmaline, Merck, Esperion, Astra Zeneca, Alnylam.
